# Analysis of different gamification-based teaching resources for physiotherapy students: a comparative study

**DOI:** 10.1186/s12909-023-04576-8

**Published:** 2023-09-18

**Authors:** Irene Sandoval-Hernández, Guadalupe Molina-Torres, Felipe León-Morillas, Carmen Ropero-Padilla, Manuel González-Sánchez, Jesús Martínez-Cal

**Affiliations:** 1 Faculty of Health Sciences, Department of Physical Therapy, University of Granada, Melilla, Spain; 2 Faculty of Health Sciences, Department of Nursing, Physiotherapy and Medicine, University of Almeria, Carretera Sacramento S/N, Almeria, 04120 Spain; 3 Faculty of Health Sciences, Department of Physiotherapy, University Catholic of Murcia – UCAM, Murcia, Spain; 4 Faculty of Health Sciences, Department of Physiotherapy, University of Malaga, Almeria, Spain; 5 Biomedical Research Institute of Malaga (IBIMA), Málaga, 29010 Spain

**Keywords:** Escape room, Gamification, Kahoot!, Physiotherapy Party, Physiotherapy

## Abstract

**Background:**

For health professionals, gamification is a new teaching method that has achieved an important role in recent years, with excellent results in learning and knowledge acquisition. Thus, the objective of this study was to analyze the gaming experience through different gamification resources in the classroom with physiotherapy students.

**Methods:**

A comparative study on gamification-based teaching resources was carried out during the first semester of the 2021–2022 academic year. A total of 33 physiotherapy students participated in this study. After the theoretical topics were taught, the participants were invited to participate in different gamification resources such as Kahoot!, Physiotherapy Party and Escape Room. The gaming experience with the different gamification resources was measured with the GAMEX scale.

**Results:**

The Physiotherapy Party showed a higher score in relation to the enjoyment dimension compared to the Kahoot! and Escape Room (p = 0.004). The Escape Room presented higher scores in absorption, creative thinking, activation and dominance compared to Kahoot! and Physiotherapy Party (p < 0.005).

**Conclusions:**

Gamification resources promote enjoyment and creativity in the students in the classroom. The use of new teaching methods based on gamification, such as Escape Room as Physiotherapy Party should be considered as first choice in the use of gamification resources due to the benefits they bring to students.

**Supplementary Information:**

The online version contains supplementary material available at 10.1186/s12909-023-04576-8.

## Background

Education for health professionals has acquired a new innovative approach in recent years [1–4]. The search for attractive and motivating training for students provides extra commitment and participation, which facilitates the achievement of academic objectives [5], leaving behind training standards in which students assume a passive and unidirectional role of knowledge integration through lectures, tutorials or laboratory practices, among others [4, 6], which are methods that can be tedious for the new generations [7]. This is partly due to the fact that the new generations have grown up surrounded by new technologies and with easy access to information, which imposes a change of direction in teaching methods and the need to make them more seductive for students and increase their commitment [2]. Student-centered teaching has proved to be efficient in learning and in retaining knowledge over time, improving skills for the development of competencies as health professionals [8]. Education and training for health professionals plays an important role in quality improvement, striving for excellence and providing safe environments where students develop the competency-based learning necessary for their professional performance [9]. In this area, the WHO states that teamwork, communication and human factors, among others, are essential criteria that must be included in the educational plans [9, 10]. Consequently, new teaching methodologies such as thought-based learning, project-based learning, flipped classroom and gamification have been incorporated [9, 11], all of them oriented towards solving problems in an autonomous, dynamic and entertaining way [6].

For health professionals, gamification is a new teaching method that has achieved an important role in recent years, with excellent results in learning and knowledge acquisition [3]. This term was integrated into the educational environment in 2010 and is used interchangeably within the literature with names such as serious games, educational games or game-based learning to describe the same concept [3, 12, 13]. However, there are nuances in the concepts, based mainly on intentionality, that may distinguish them [3, 14]. More specifically, gamification is described as the application of game designs in contexts other than gaming [4, 5, 13, 15], a generically used concept that, in the educational environment, refers to learning through methodology and game mechanics [7, 12, 13]. Far from the playful nature of games, gamification in the education of health professionals bases its premises on specific game elements: own language, challenge; problem; conflict; critical reasoning; environment; staging; teamwork; negotiation; leadership; rules; evaluation; points; goals; objectives; and rewards [3, 16]. These can be applied to the guidelines implicit in the acquisition of knowledge through the cognitive process: remember, understand, apply, analyze, evaluate and create [16]. These game attributes, i.e., challenges, interactivity, rules, scoring and teamwork [5], are used in order to fully involve the students through practice [17], encouraging motivation, interaction, critical thinking and promoting the acquisition and evaluation of knowledge and skills [12, 18], in addition to involving students in learning and fostering the capacity for commitment [14]. Gamification as an educational tool contributes to student-teacher feedback and the feedback generated by this tool provides relevant information about the scope of content assimilation, being useful for the evaluation of acquired knowledge [19].

Different studies have explored the influence of game-based teaching methods on university degrees of health sciences, mainly in Nursing, as a complement to traditional teaching, in order to strengthen the skills and knowledge acquired for handling realistic situations with patients [20, 21]. The effects of these teaching tools and what methods are involved in them have also been analyzed [3]. However, few studies are focused on other health professions, such as Physiotherapy, only four studies have been found concerning the use of gamification resources as a method of teaching and learning in Physiotherapy students, where they have been compared with traditional methods, analyzing the influence of the acquisition of competencies and student satisfaction describing the beneficial use compared to traditional methods in terms of retention and assimilation of content and motivation of students [22–25]. However, there are no studies that compare which gamification modality provides greater satisfaction in the learning process. Therefore, the objective of this study was to analyze the gaming experience through different gamification resources Kahoot!, Physiotherapy Party and Escape Room in the classroom with Physiotherapy students using the GAMEX scale.

## Methods

### Study design

During the first semester of the 2021–2022 academic year, a quantitative study was carried out, in which different gamification resources were compared in the classroom through game-based learning in Physiotherapy Degree students. Convenience sampling was performed. A total of 33 students participated in the Physiotherapy First Aid subject, including three gamification resources, such as Kahoot [26]!, Physiotherapy Party [25] and Escape Room [2]. The different gamification resources were compared using the validated GAMEX gaming experience scale [27].

### Setting and participants

**Fig. 1:**
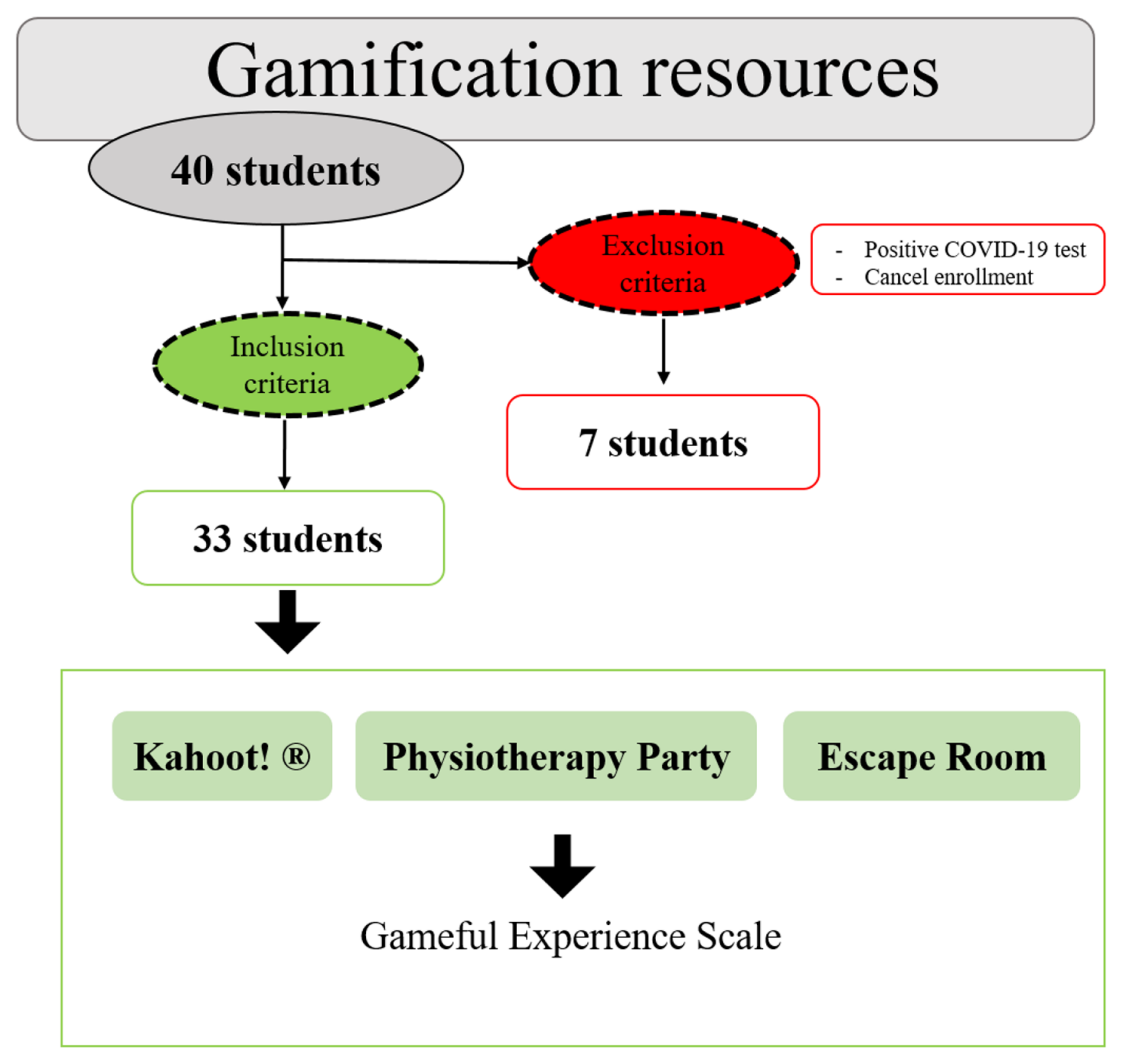
Flow diagram of participants

This study was carried out at the University of Almeria. The initial participants in the study were 40 students of the Physiotherapy Degree enrolled in the subject “First Aid from Physiotherapy”, which is an optional subject of 6 European Credit Transfer and Accumulation System (ECTS) credits, and is taught in the first semester of the 4th year of the Physiotherapy degree. This subject consists in the acquisition of practical skills and theoretical content, where students are organized in groups of 15–20 students, depending on the number of students enrolled, to develop practical content. The theoretical-practical contents of this subject introduce students to the generalities of First Aid from Physiotherapy, basic cardiopulmonary resuscitation, airway obstruction, trauma injuries, hemorrhage, skin aggression, emergency delivery, alterations of consciousness, aggression by physical or environmental agents and intoxication. (Supplementary file 1) The inclusion criteria were the following: (a) being over 18 years old, and (b) being enrolled in the elective subject of First Aid from Physiotherapy. On the other hand, the exclusion criteria were the following: (a) positive COVID-19 test, which made it impossible to participate in the classes where each of the gamification resources were developed (Kahoot!, Physiotherapy Party and Escape Room), and (b) cancelation of enrollment in the subject during the semester (Fig. 1). The study was developed in the groups constituted for the practical classes, of obligatory attendance established by the academic plans of the university, to ensure the attendance of the students.

### Instruments

After collecting the information regarding the sociodemographic characteristics of the students, the following measurement instrument was used:

#### Gaming experience scale (GAMEX)

**Fig. 2:**
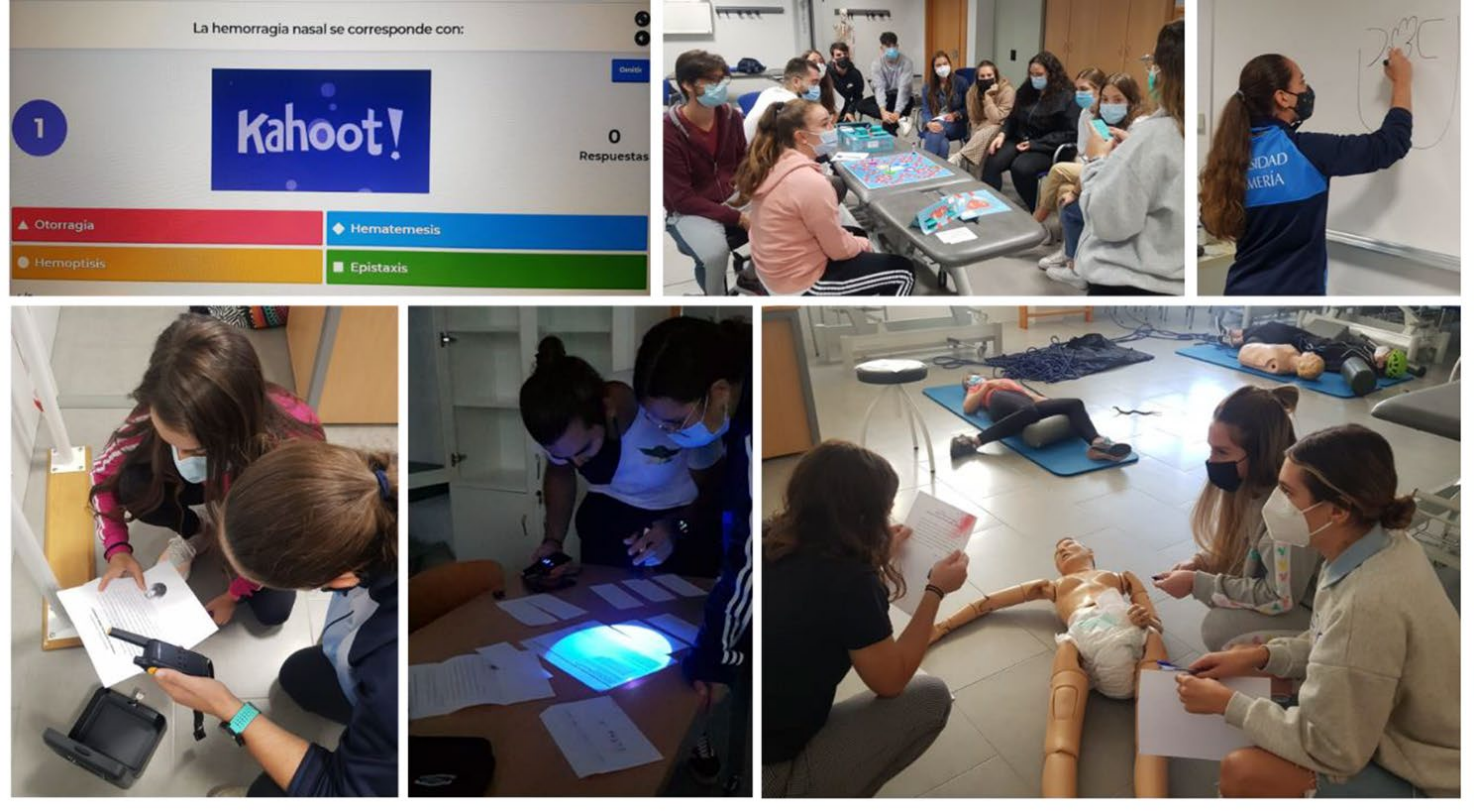
Kahoot!, Physiotherapy Party and Escape Room

It measures the gaming experience among Physiotherapy students during the Escape Room [27, 28]. It consists of 27 items, which are scored using a Likert scale, with a range from 1 (never) to 5 (always). These 27 items are divided into 6 dimensions: enjoyment, absorption, creative thinking, activation, absence of negative effects, and dominance. The total Cronbach’s α value was 0.855 [27]. The gamification resources used to carry out this study are detailed below (Fig. 2).

#### Kahoot!

Kahoot! is a real-time platform for game-based learning. It is a free formative assessment resource that is widely used in education [26, 29]. Kahoot! allows teachers to create four different types of games, only quizzes and true-false questions were used, where participants compete against each other. The teacher can track responses in real time. High scores are returned for each question, and overall winners are displayed on a scoreboard at the end of the session [30]. A Kahoot! was performed at the end of each topic of the theoretical content. The students were divided into groups of 4–5 people to compete in the game. Groups of 4–5 students were set up. The total time spent on this resource was 1 h in all. (Supplementary file 2)

#### Physiotherapy party

The game “Physiotherapy Party – Guadaña & CO” was used [25]. The objective of this gamification resource is for the participants to win each of the different challenges in mimicry, questions, forbidden words and drawings about the contents of the subject in order to obtain scores with each card and obtain each of the tokens from the main squares, and then carry out the final test. The students were divided into groups of 4–5 people to compete in the game. These students had to show their knowledge acquired in the theoretical part of the course. The cards were made by the students during the course as they completed the course topics. The total time spent on this resource was 1 h. (Supplementary File 3)

#### Escape room

In this game, the participants are introduced to a scenario where they must solve puzzles and use clues to complete the activity and thus escape from the room within a certain amount of time [31]. The students were divided into groups of 4–5 people, using two different rooms. To solve the clinical case, they had to communicate by walkie talky and complete all the tests. Among the tests, they had to decipher a hidden message with ultraviolet light, open locks and safes and correctly carry out the first-aid protocols learned in the subject. The total time spent on this resource was 1 h. (Supplementary File 4)

### Procedure

Throughout the semester, different tests of Kahoot! were performed at the end of each topic of the theoretical part of the subject, collecting information regarding the gaming experience with Kahoot!, using the GAMEX scale. At the end of the Physiotherapy Party – Guadaña & CO, information regarding the GAMEX scale was also collected. Finally, after the completion of the Escape Room, the information related to the GAMEX scale was also collected.

### Data analysis

Firstly, a descriptive analysis of the results was carried out, calculating the measures of central tendency and dispersion for the quantitative variables, while, for the categorical variables, the frequency and percentage were analyzed. For the comparisons between the three gamification tools, an ANOVA analysis was carried out, to analyze the differences between groups and within the group, specifying the multiple comparisons with the Bonferroni test. A value of p < 0.05 was considered significant. For data analysis, the statistical software SPSS version 25 was used.

### Ethical considerations

The students were informed about the purpose of the study, and about the confidentiality and anonymous treatment of the data. Before data collection, the study was approved by the Ethics Committee of the University of Almeria (EFM 156/2021), and the data was used in accordance with Organic Law 3/2018, of December 5, of Protection of Personal Data and guarantee of digital rights. The ethical principles established in the Declaration of Helsinki were also followed. Subsequently, the participants signed the informed consent.

## Results

### Sociodemographic characteristics of the participants

The sample consisted of 40 Physiotherapy Degree students, of which 33 students met the inclusion criteria, thus participating through the three gamification-based teaching resources. Of the total sample included in the study, 17 were women (51.5%) and 16 men (48.5%), with a mean age of 21.48 ± 1.73 years (women: 21.35 ± 1.22 years, between 20 and 25 years; men: 21.62 ± 2.18 years, between 20 and 29 years). One hundred per cent of the study participants were taking the subject “First Aid from Physiotherapy” and had not previously used gamifying resources for learning.

### Game experience scale (GAMEX) regarding Kahoot!, Physiotherapy Party and escape room

The results obtained in the game experience scale are detailed in Table 1, where the scores obtained in each of the GAMEX dimensions are specified in relation to Kahoot!, Physiotherapy Party and Escape Room over the total number of participants. After carrying out the ANOVA analysis to compare the different gamification resources, the results of the inter-group and intra-group differences were obtained (Table 2); Table 2 presents the multiple comparisons with respect to the three gamification resources.

**Table 1:** Kahoot!, Physiotherapy Party and Escape Room – Gameful Experience GAMEX

**Dimension** (Range)	**Kahoot!** M ± SD	**Physiotherapy Party** M ± SD	**Escape Room** M ± SD	**F**	***p*** **value**
**Enjoyment** (6–30)	26,24 ± 3,58	28,12 ± 5,29	27,67 ± 6,81	5,95	**0,004**
**Absorption** (6–30)	21,33 ± 6,12	23,18 ± 6,96	25,52 ± 5,73	6,66	**0,002**
**Creative thinking** (4–20)	13,48 ± 4,48	16,45 ± 4,10	17,61 ± 4,13	16,86	**0,000**
**Activation** (4–20)	15,42 ± 2,96	15,27 ± 4,11	15,61 ± 3,88	0,39	0,676
**Absence of negative effects** (3–15)	3,91 ± 1,48	4,00 ± 2,06	3,18 ± 1,04	2,70	0,072
**Dominance** (4–20)	14, 76 ± 3,23	15,27 ± 3,85	16,09 ± 4,03	3,27	**0,042**

**Bold p value:** *statistically significant differences*

**Table 2:** Multiple comparison between gamification-based teaching resources

Dimension	(I) Gameful Tool	(J) Gameful Tool	Difference of means (I-J)	Deviation Error	*p* value
GAMEX enjoyment	Kahoot!	Physiotherapy Party	-2,788	0,867	**0,005**
		Escape Room	-2,333	0,867	**0,025**
	Physiotherapy Party	Kahoot!	2,788	0,867	**0,005**
		Escape Room	0,455	0,867	1,000
	Escape Room	Kahoot!	2,333	0,867	**0,025**
		Physiotherapy Party	-0,455	0,867	1,000
GAMEX absorption	Kahoot!	Physiotherapy Party	-2,758	1,301	0,110
		Escape Room	-4,727	1,301	**0,001**
	Physiotherapy Party	Kahoot!	2,758	1,301	0,110
		Escape Room	-1,970	1,301	0,400
	Escape Room	Kahoot!	4,727	1,301	**0,001**
		Physiotherapy Party	1,970	1,301	0,400
GAMEX creative thinking	Kahoot!	Physiotherapy Party	-3,576	0,849	**0,000**
		Escape Room	-4,727	0,849	**0,000**
	Physiotherapy Party	Kahoot!	3,576	0,849	**0,000**
		Escape Room	-1,152	0,849	0,534
	Escape Room	Kahoot!	4,727	0,849	**0,000**
		Physiotherapy Party	1,152	0,849	0,534
GAMEX activation	Kahoot!	Physiotherapy Party	-0,273	0,720	1,000
		Escape Room	-0,636	0,720	1,000
	Physiotherapy Party	Kahoot!	0,273	0,720	1,000
		Escape Room	-0,364	0,720	1,000
	Escape Room	Kahoot!	0,636	0,720	1,000
		Physiotherapy Party	0,364	0,720	1,000
GAMEX absence of negative effects	Kahoot!	Physiotherapy Party	-0,182	0,369	1,000
		Escape Room	0,636	0,369	0,264
	Physiotherapy Party	Kahoot!	0,182	0,369	1,000
		Escape Room	0,818	0,369	0,087
	Escape Room	Kahoot!	-0,636	0,369	0,264
		Physiotherapy Party	-,818	0,369	0,087
GAMEX dominance	Kahoot!	Physiotherapy Party	-1,091	0,726	0,408
		Escape Room	-1,848	0,726	**0,037**
	Physiotherapy Party	Kahoot!	1,091	0,726	0,408
		Escape Room	-0,758	0,726	0,897
	Escape Room	Kahoot!	1,848	0,726	**0,037**
		Physiotherapy Party	0,758	0,726	0,897

According to the obtained results, there were higher scores in the enjoyment dimension for Physiotherapy Party compared to Kahoot! and Escape Room. The highest scores regarding absorption, creative thinking, activation and dominance were obtained in Escape Room with respect to Kahoot! and Physiotherapy Party. At the same time, the lowest scores regarding the absence of negative effects were also obtained in Escape Room. There were statistically significant differences between the different gamification resources in terms of the GAMEX dimensions of enjoyment, absorption, creative thinking and dominance.

As can be seen in Table 2, there are statistically significant differences in different dimensions of the GAMEX scale, such as enjoyment, absorption, creative thinking and dominance. In the enjoyment dimension, differences exist between Physiotherapy Party and Escape Room with respect to Kahoot!, as well as in creative thinking. On the other hand, in the absorption dimension, the differences exist between Escape Room and Kahoot!. Lastly, in the dominance dimension, there were differences between Escape Room and Kahoot!. It should be noted, as can be seen in Table 1, that the GAMEX scale scores in the dimensions that showed statistically significant differences were higher in both Physiotherapy Party and Escape Room compared to Kahoot!.

## Discussion

The objective of this study was to analyze the gaming experience through different gamification resources in the classroom with Physiotherapy students. Once the results were analyzed, it was observed that there were statistically significant differences with respect to the GAMEX game experience scale in relation to the dimensions of enjoyment, absorption, creative thinking and dominance between the three gamifying resources used in this study in relation to Kahoot!, Physiotherapy Party and Escape Room. However, these results cannot be compared in terms of gaming experience scale, since there is no study where the GAMEX scale has been used. However, Kahoot! is widely used in several educational fields, such as nursing [32, 33], medicine [34, 35], dentistry [36], histology and cell biology lab learning sessions [37], pharmacology [38, 39] and computer engineering [40]. In addition, the learning results are satisfactory and the students are precursors of the use of Kahoot! in class due to the increased motivation and commitment it generates towards the subject [35]. Therefore, the use of Kahoot! as a gamification resource in the classroom promotes active learning, increases enjoyment and participation, provides feedback [29], improves performance [40] and stimulates learning [36].

Furthermore, the game experience scale in relation to the Physiotherapy Party can be compared with another study [25], where similar results were obtained in each of the GAMEX dimensions; however, there is no other study comparing the Physiotherapy Party game experience with other gamification resources. At the same time, in relation to the use of the Escape Room in the classroom, it can be said that there is no other study that compares this gamification resource with others. Therefore, the Escape Room can be considered as an attractive resource in the teaching-learning process by students [4, 9, 18, 20, 31, 41, 42], but also as an evaluation method [11], obtaining similar results in each of the GAMEX dimensions [8, 11]. Thus, this gamification resource is effective both in the teaching-learning process and in the evaluation process [11].

In this scenario, the fact that the different methods in the teaching-learning process using the gamification resources of this study show positive results compared to traditional methods has great weight in the literature, however, comparisons with other resources have not been investigated previously, so it is necessary to seek an explanation for the differences in the results of this study. The GAMEX scale with which Kahoot!, Physiotherapy Party and Escape Room have been analyzed, presents different dimensions in which statistically significant differences between one method and the other have been highlighted [12, 27]. The enjoyment dimension acquired better results from Escape Room and Physiotherapy Party versus Kahoot! This may be due to the fact that these two resources have an environment similar to that of a playful game, so students could play this game without being part of a teaching-learning methodology. They experienced pleasant emotions, answering positively to all the items where they found it fun, entertaining, they liked playing, they answered positively to the items that made reference to it [43]. In the creative thinking dimension, the items referring to this dimension stood out positively; in this sense, Escape Room and Physiotherapy Party obtained better results, showing statistical significance. The items refer to the adventurous spirit, the awakening of the imagination, creativity and the ability to explore the topics covered. It could be that the fact of experiencing positive emotions brings with it an increase in students’ involvement in all aspects related to this dimension [44]. On the other hand, Escape Room stood out in a statistically significant way with respect to Kahoot! in the dimensions of absorption and mastery; absorption includes elements such as full concentration on what you are doing, without remembering what surrounds the game and losing track of time, requiring full attention to the scenario where they develop. Mastery refers to the sense of responsibility and leadership, as well as autonomy and self-confidence, competencies necessary for the professional development of health professionals [45]. In this sense, these two resources are completely different, in Escape Room there is an environment created from a story or narrative, it is necessary to put all the senses to, in addition to solving questions or tests, discover which is the next and so on until you reach a final goal. In the case of Kahoot! It is possible that the questions are similar, since they are contents of the same subject, but they are carried out from a more passive participation, without elements that can stimulate [46].

As we have seen, these gamification resources present beneficial characteristics in the competency-based learning process [5, 9, 12, 17, 18, 20, 35, 47], but also in evaluation processes [8, 11, 21, 33, 37, 39–41]. In the case of this study, Physiotherapy Party and Escape Room are the gamification resources with the best results in the game experience, but more comparative studies between gamification resources should be done since this study has some limitations.

### Strengths, limitations and future lines of research

It should be noted that there are no previous studies carried out with Physiotherapy Degree students comparing different gamification resources in the classroom, such as Kahoot!, Physiotherapy Party and Escape Room. The results of this study should be considered in the context of several limitations. Firstly, the students who participated in this study were from a single Spanish university and from a single year, and the sample of students is small, since the participants were from an optional subject. Secondly, the degree of satisfaction, usefulness and effort involved in the use of the different gamification resources by teachers was not measured, which would have allowed us to obtain an even deeper understanding of their level of satisfaction with this type of gamification-based teaching resources. Further research should be carried out to measure the impact of the use of this type of resources and thus allow planning the teaching-learning process using all available tools.

## Conclusions

The main conclusion that can be drawn from this study is that, through Physiotherapy Party and Escape Room as gamification resources in the teaching process in Physiotherapy students, students present higher levels of enjoyment and creative thinking with respect to the use of Kahoot!. In addition, the absorption and dominance levels are also higher when using Escape Room compared to Kahoot!. Therefore, the results suggest that both Escape Room and Physiotherapy Party should be considered as the first choice in the use of gamification resources due to the benefits they bring to Physiotherapy Degree students.

### Electronic supplementary material

Below is the link to the electronic supplementary material.

Supplementary Material 1

## Data Availability

The data that support the findings of this study are available on request from the corresponding author. The data are not publicly available due to privacy or ethical restrictions.

## References

[1] Bharamgoudar R (2018). Gamification Clin Teach.

[2] Guckian J, Eveson L, May H (2020). The great escape? The rise of the escape room in medical education. Futur Healthc J.

[3] van Gaalen A, Brouwer J, Schönrock-Adema J, Bouwkamp-Timmer T, Jaarsma A, Georgiadis J (2021). Gamification of health professions education: a systematic review. Adv Heal Sci Educ.

[4] Kaul V, Morris A, Chae JM, Town JA, Kelly WF. Delivering a Novel Medical Education “ Escape Room ” at a National Scientific Conference: First Live, Then Pivoting to Remote Learning Because of COVID-19. Chest. 2021;160:1424–32. 10.1016/j.chest.2021.04.069 PMC8457933 34029564

[5] Woolwine S, Jackson B (2019). Game On.

[6] Sahu PK, Chattu VK, Rewatkar A, Sakhamuri S (2019). Best practices to impart clinical skills during preclinical years of medical curriculum. J Educ Health Promot.

[7] Brull S, Finlayson S (2016). Importance of Gamification in increasing learning. J Contin Educ Nurs.

[8] Molina-Torres G, Sandoval-Hernández I, Ropero-Padilla C, Rodriguez-Arrastia M, Martínez-Cal J, Gonzalez-Sanchez M (2021). Escape Room vs. Traditional Assessment in Physiotherapy Students’ anxiety, stress and gaming experience: a comparative study. Int J Environ Res Public Health.

[9] Backhouse A, Malik M (2019). Escape into patient safety: bringing human factors to life for medical students. BMJ Open Qual.

[10] Maheu-Cadotte MA, Cossette S, Dubé V, Fontaine G, Mailhot T, Lavoie P et al. Effectiveness of serious games and impact of design elements on engagement and educational outcomes in healthcare professionals and students: a systematic review and meta-analysis protocol. BMJ Open. 2018;8. 10.1136/bmjopen-2017-019871 PMC5857654 29549206

[11] Gutiérrez-Puertas L, Márquez-Hernández VV, Román-López P, Rodríguez-Arrastia MJ, Ropero-Padilla C, Molina-Torres G (2020). Escape rooms as a clinical evaluation method for nursing students. Clin Simul Nurs.

[12] Sera L, Wheeler E (2017). Game on: the gamification of the pharmacy classroom. Curr Pharm Teach Learn.

[13] Rutledge C, Walsh C, Swinger N, Auerbach M, Castro D, Dewan M (2018). Gamification in action: theoretical and practical considerations for medical educators. J Assoc Am Med Coll.

[14] Ahmed A, Sutton MJD (2017). Gamification, serious games, simulations, and immersive learning environments in knowledge management initiatives. World J Sci Technol Sustain Dev.

[15] Donkin R, Rasmussen R (2021). Student Perception and the effectiveness of Kahoot!: a scoping review in histology, anatomy, and Medical Education. Anat Sci Educ.

[16] Echeverría A, García-Campo C, Nussbaum M, Gil F, Villalta M, Améstica M (2011). A framework for the design and integration of collaborative classroom games. Comput Educ.

[17] Eukel HN, Frenzel JE, Cernusca D (2017). Educational Gaming for Pharmacy Students - Design and evaluation of a diabetes-themed escape room. Am J Pharm Educ.

[18] Anguas-Gracia A, Subirón-Valera AB, Antón-Solanas I, Rodríguez-Roca B, Satústegui-Dordá PJ, Urcola-Pardo F (2021). An evaluation of undergraduate student nurses’ gameful experience while playing an escape room game as part of a community health nursing course. Nurse Educ Today.

[19] Abdulmajed H, Park YS, Tekian A (2015). Assessment of educational games for health professions: a systematic review of trends and outcomes. Med Teach.

[20] Gómez-Urquiza JL, Gómez-Salgado J, Albendín-García L, Correa-Rodríguez M, González-Jiménez E (2019). Cañadas-De la Fuente GA. The impact on nursing students’ opinions and motivation of using a “Nursing escape Room” as a teaching game: a descriptive study. Nurse Educ Today.

[21] Roman P, Rodriguez-Arrastia M, Molina-Torres G, Márquez-Hernández VV, Gutiérrez-Puertas L, Ropero-Padilla C (2020). The escape room as evaluation method: a qualitative study of nursing students’ experiences. Med Teach.

[22] Chong DYK (2019). Benefits and challenges with gamified multi-media physiotherapy case studies: a mixed method study. Arch Physiother.

[23] Cortés-Pérez I, Zagalaz-Anula N, López-Ruiz M, del C, Díaz-Fernández Á, Obrero-Gaitán E, Osuna-Pérez MC. Study based on gamification of tests through Kahoot!™ and reward game cards as an innovative Tool in Physiotherapy students: a preliminary study. Healthc. 2023;11. 10.3390/healthcare11040578 PMC9957048 36833112

[24] Ferrer-Sargues FJ, Kot Baixauli PE, Carmenate-Fernández M, Rodríguez-Salvador G, González Domínguez J, Martínez-Olmos FJ et al. Escape-cardio: Gamification in cardiovascular physiotherapy. An observational study. Nurse Educ Today. 2021;106 July. 10.1016/j.nedt.2021.105062 34304100

[25] Molina-Torres G, Rodriguez-Arrastia M, Alarcón R, Sánchez-Labraca N, Sánchez-Joya M, Roman P (2021). Game-based learning outcomes among physiotherapy students: comparative study. JMIR Serious Games.

[26] Kahoot!. 2013. https://kahoot.com/.

[27] Márquez-Hernández VV, Garrido-Molina JM, Gutiérrez-Puertas L, García-Viola A, Aguilera-Manrique G, Granados-Gámez G (2019). How to measure gamification experiences in nursing? Adaptation and validation of the Gameful Experience Scale [GAMEX]. Nurse Educ Today.

[28] Eppmann R, Bekk M, Klein K (2018). Gameful Experience in Gamification: construction and validation of a Gameful Experience Scale [GAMEX]. J Interact Mark.

[29] Ismail MA, Ahmad A, Mohammad JA, Fakri N, Nor M, Pa M (2019). Using Kahoot! As a formative assessment tool in medical education: a phenomenological study. BMC Med Educ.

[30] Cugelman B, Gamification (2013). What it is and why it matters to digital health behavior change developers. JMIR Serious Games.

[31] Adams V, Burger S, Crawford K, Setter R (2018). Can you escape? Creating an escape room to facilitate active learning. J Nurses Prof Dev.

[32] Aras GN, Çiftçi B (2021). Comparison of the effect of reinforcement with question-answer and kahoot method on the success and motivation levels of nursing students: a quasi-experimental review. Nurse Educ Today.

[33] Öz G, Ordu Y. The effects of web based education and kahoot usage in evaluation of the knowledge and skills regarding intramuscular injection among nursing students. Nurse Educ Today. 2021;103 April. 10.1016/j.nedt.2021.104910 34000592

[34] Jamil Z, Fatima SS, Saeed AA (2018). Preclinical medical students’ perspective on technology enhanced assessment for learning. J Pak Med Assoc.

[35] Neureiter D, Klieser E, Neumayer B, Winkelmann P, Urbas R, Kiesslich T (2020). Feasibility of kahoot! As a real-time assessment tool in (Histo-)pathology classroom teaching. Adv Med Educ Pract.

[36] Felszeghy S, Pasonen-Seppanen S, Koskela A, Nieminen P, Harkonen K, Paldanius KMA (2019). Using online game-based platforms to Improve Student Performance and Engagement in Histology Teaching. BMC Med Educ.

[37] Kalleny N (2020). Advantages of Kahoot! Game-based formative assessments along with methods of its use and application during the COVID-19 pandemic in various live learning sessions. J Microsc Ultrastruct.

[38] Bryant SG, Correll JM, Clarke BM (2018). Fun with pharmacology: winning students over with kahoot! Game-based learning. J Nurs Educ.

[39] Sumanasekera W, Turner C, Ly K, Hoang P, Jent T, Sumanasekera T (2020). Evaluation of multiple active learning strategies in a pharmacology course. Curr Pharm Teach Learn.

[40] Fuster-Guilló A, Pertegal-Felices ML, Jimeno-Morenilla A, Azorín-López J, Rico-Soliveres ML, Restrepo-Calle F (2019). Evaluating impact on motivation and academic performance of a game-based learning experience using Kahoot. Front Psychol.

[41] Cole JD, Ruble MJ (2021). Designing and evaluating game-based learning for continuing pharmacy education using an “escape room” activity. Curr Pharm Teach Learn.

[42] López-Belmonte J, Segura-Robles A, Fuentes-Cabrera A, Parra-González ME. Evaluating activation and absence of negative effect: gamification and escape rooms for learning. Int J Environ Res Public Health. 2020;17. 10.3390/ijerph17072224 PMC7177750 32224978

[43] Perdomo Vargas IR, Rojas Silva JA (2019). La ludificación como herramienta pedagógica: algunas reflexiones desde la psicología. Rev Estud y Exp en Educ.

[44] Anzelin I, Marín-Gutiérrez A (2020). Relación entre la emoción y los procesos de enseñanza aprendizaje. Sophia.

[45] Márquez UC, Fasce HE, Pérez VC, Ortega BJ, Parra PP, Ortiz ML (2014). Aprendizaje autodirigido y su relación con estilos y estrategias de aprendizaje en estudiantes de medicina. Rev Med Chil.

[46] Flores-Sierra E (2016). Development of attention and its involvement in learning process. Rev Didasc@lia D&E.

[47] Brull S. Importance of Gamification in increasing learning. 2012. 10.3928/00220124-20160715-09. 10.3928/00220124-20160715-09 27467313

