# Supplementary material for: Analysis of different gamification-based teaching resources for physiotherapy students: a comparative study
Source: BMC Med Educ. 2023 Sep 18;23:675. doi: 10.1186/s12909-023-04576-8 (PMC10506183; doi:10.1186/s12909-023-04576-8)

**SUPPLEMENTARY FILE 1. EDUCATIONAL THEMES, COMPETENCES AND LEARNING OBJECTIVES OF THE SUBJECT "FIRST AID FROM PHYSIOTHERAPY".**


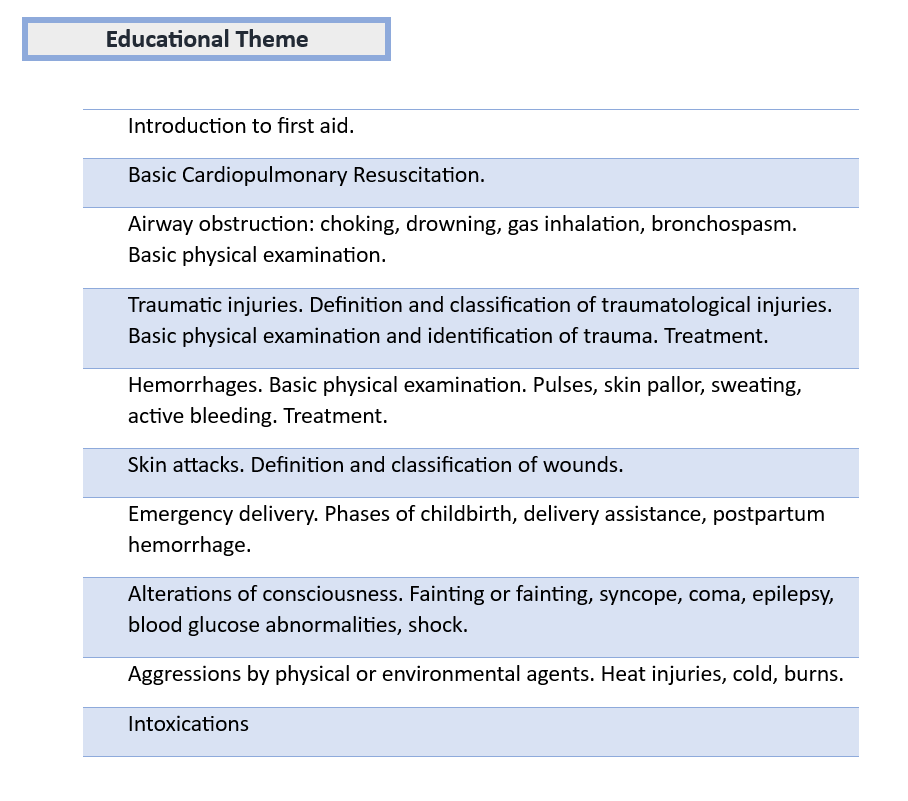


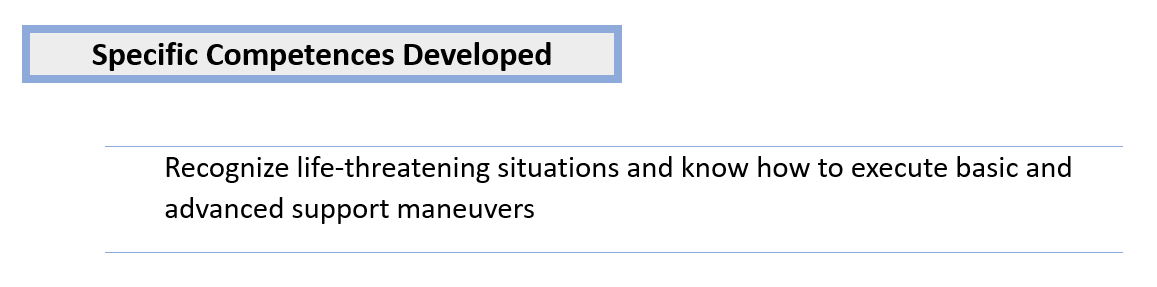


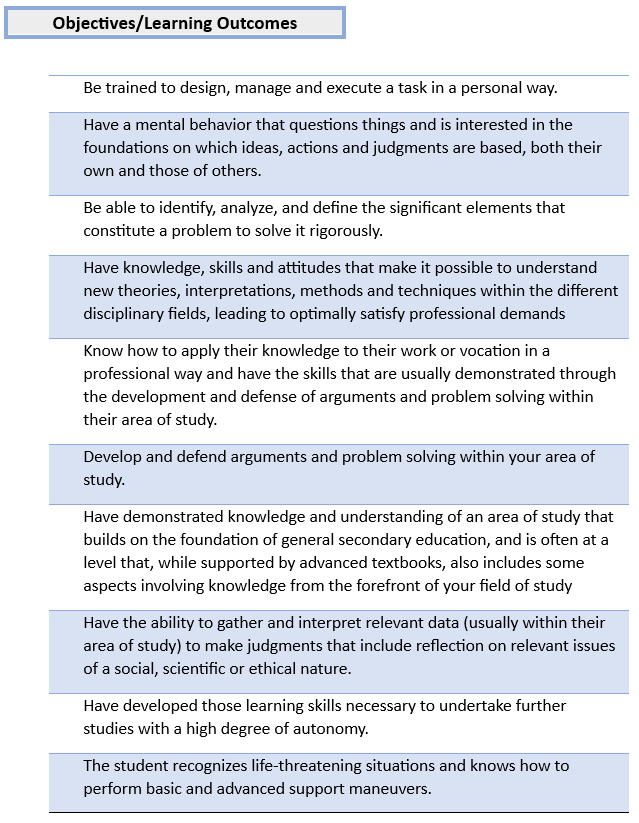


**SUPPLEMENTARY FILE 2. KAHOOT! APPLIED TO THE SUBJECT “FIRST AID FROM PHYSIOTHERAPY”**

1.
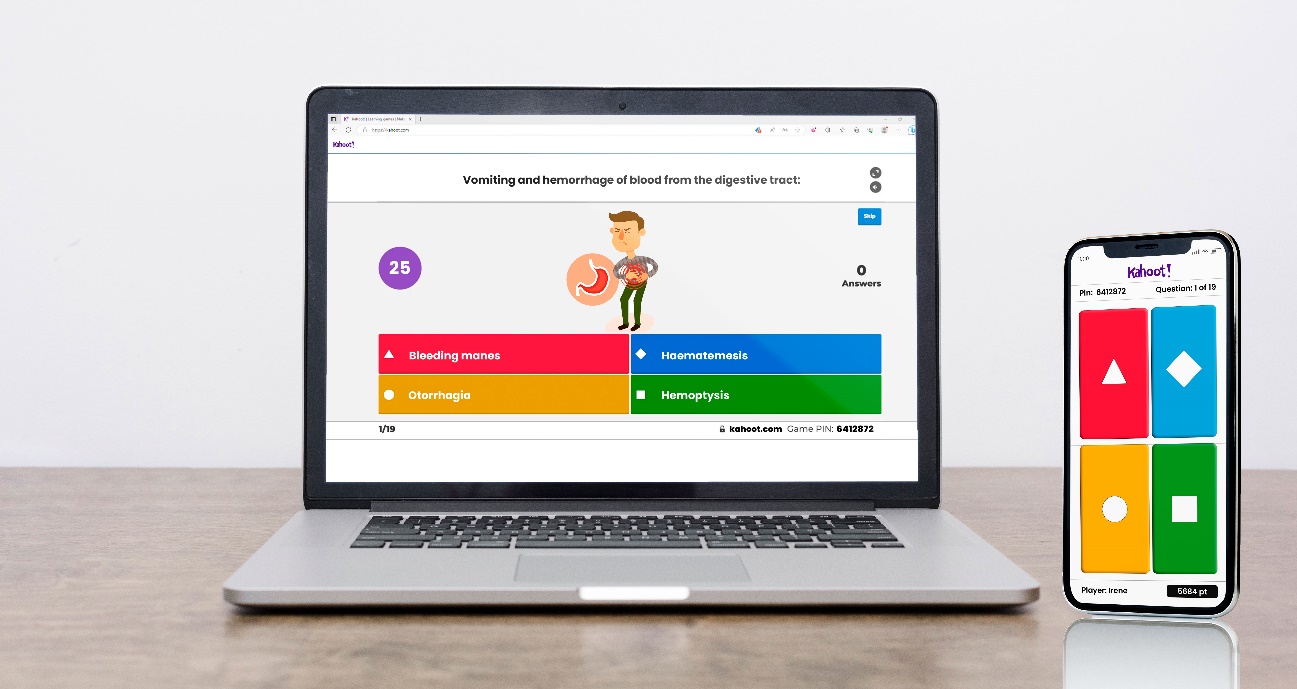
Illustration about Kahoot! in Quiz mode
2. Illustration about Kahoot! in True/False mode


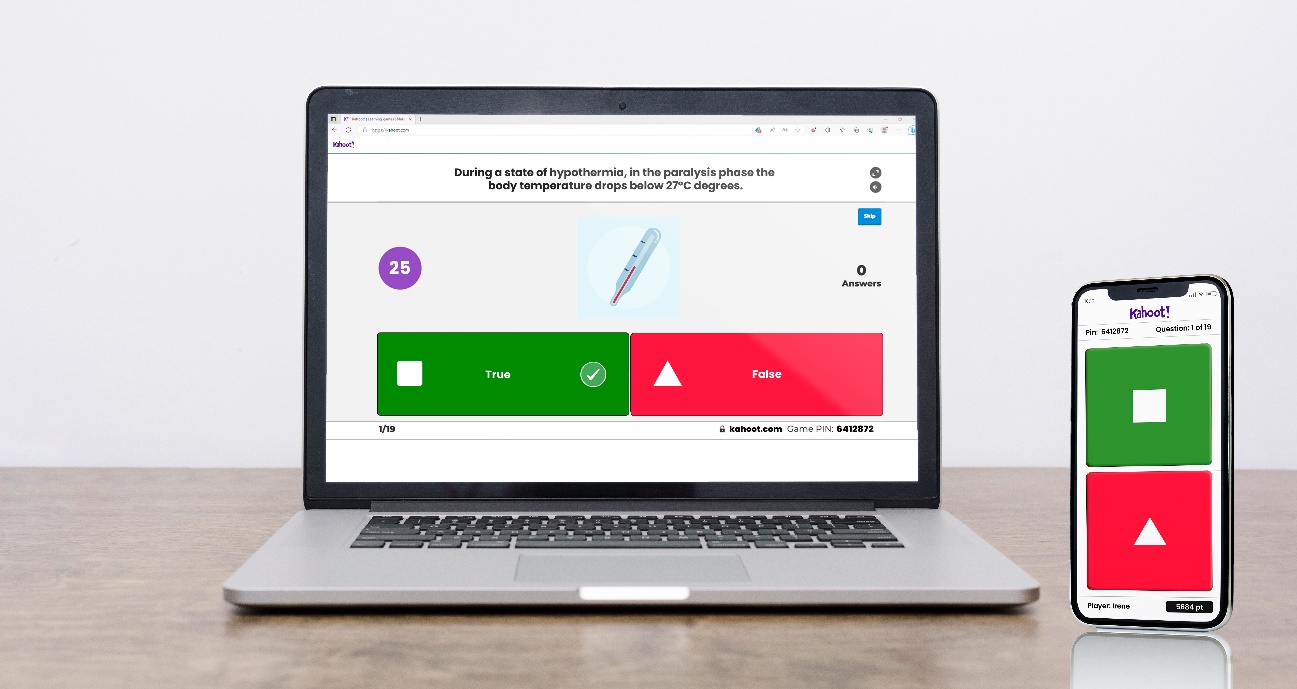


**SUPPLEMENTARY FILE 3. PHYSIOTHERAPY PARTY APPLIED TO THE SUBJECT “FIRST AID FROM PHYSIOTHERAPY”**

1. Elements which set up “Physiotherapy Party Game.”


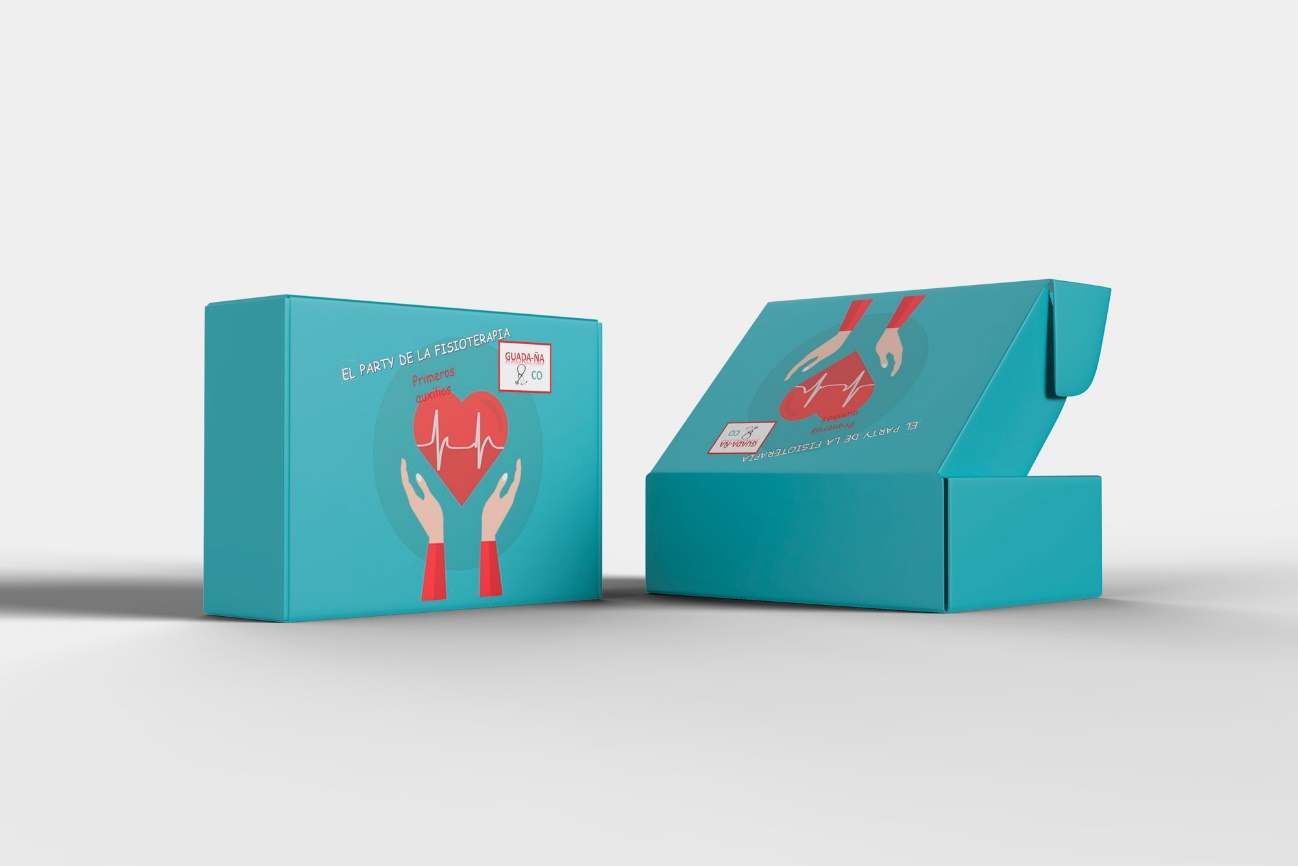


**
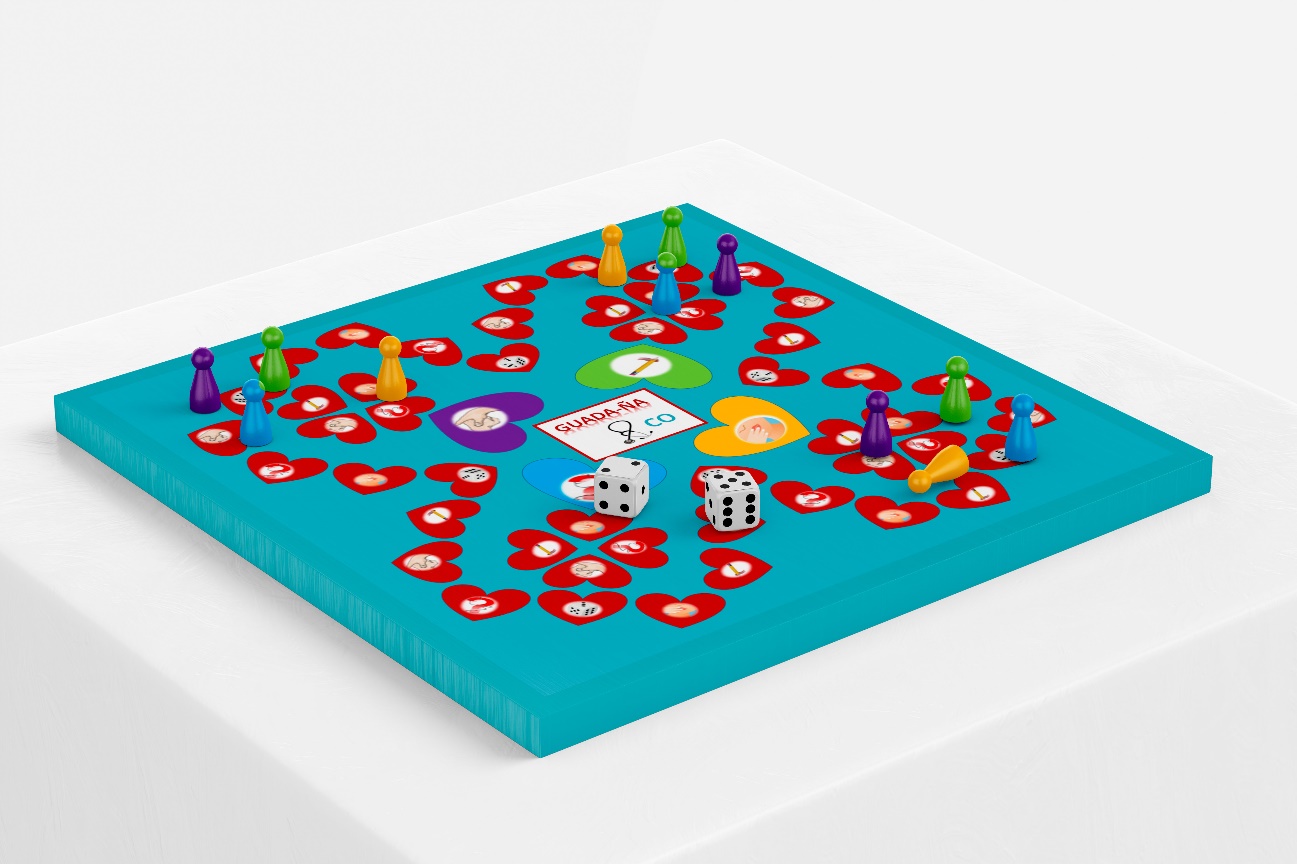
**


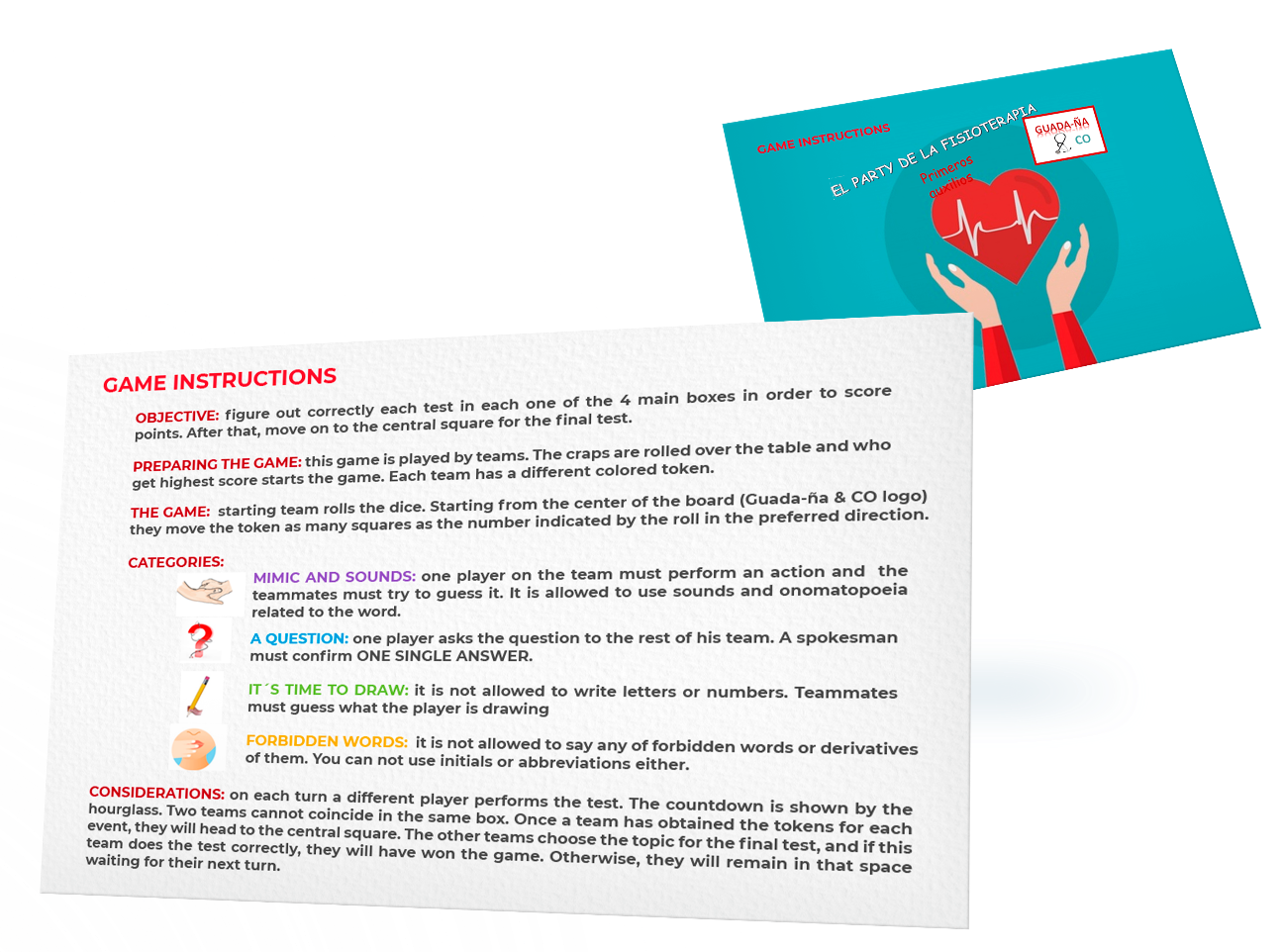


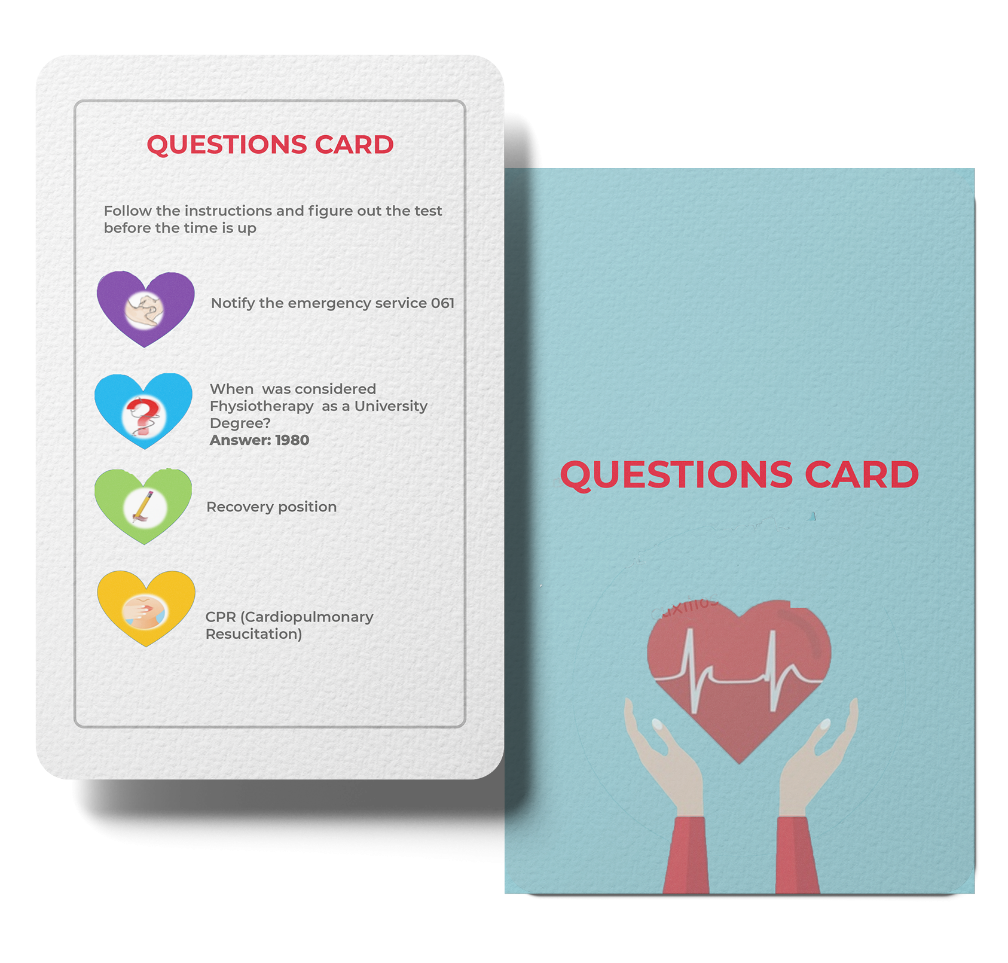


**SUPPLEMENTARY FILE 4. ESCAPE-ROOM APPLIED TO THE SUBJECT “FIRST AID FROM PHYSIOTHERAPY”**

1. Illustration about Escape-Room
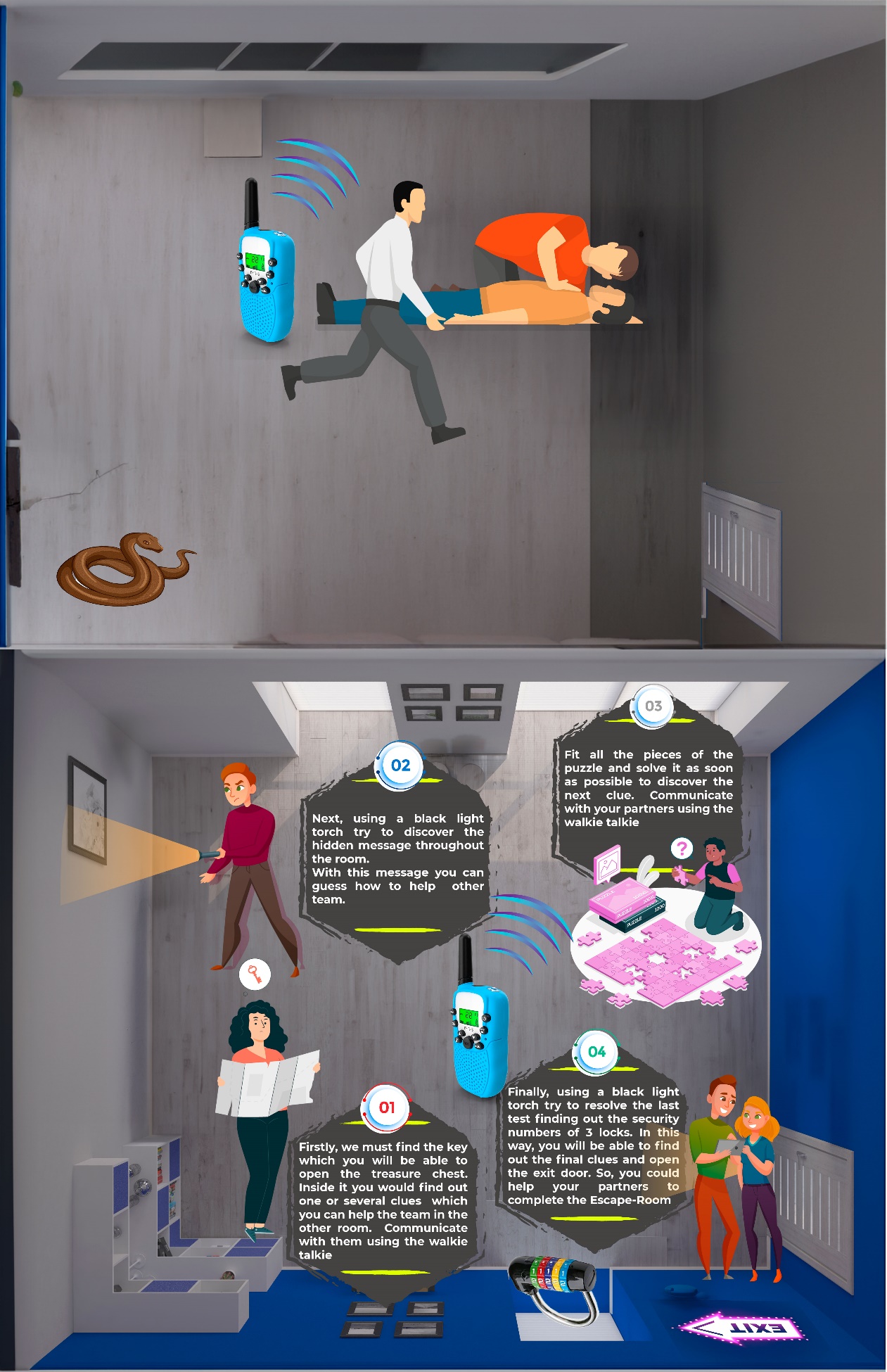

2. Main target of the game

The main objective of the game is the next: working as a team and following the instructions obtained after passing the different tests, try to complete the game by solving the clinical case: saving the life of a climber who has fallen after a snake has bitten him.

In this way, everyone will be able to get out the rooms and complete the Escape Room Game.

1. Items


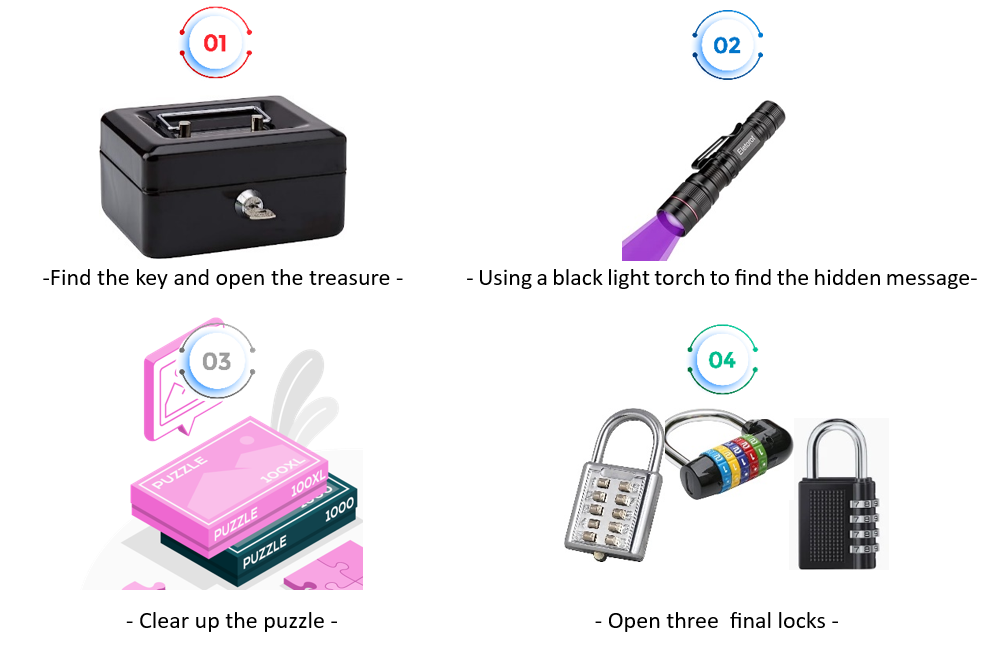


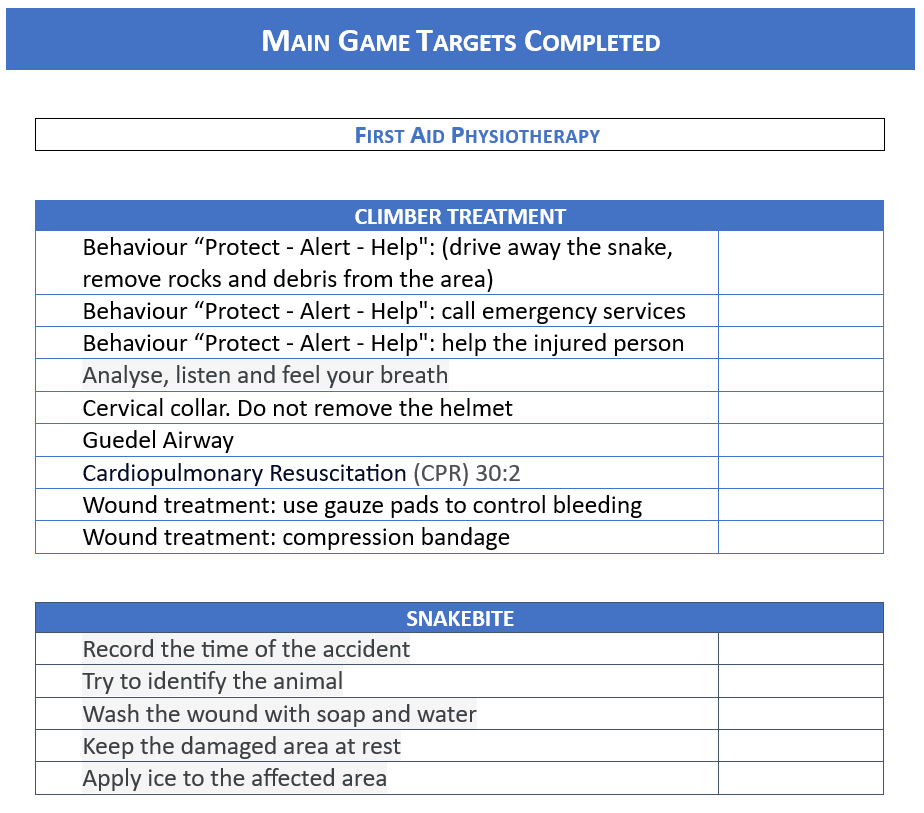

Supplement: Supplementary file 1 — Supplementary Material 1 [file 12909_2023_4576_MOESM1_ESM.docx]
